# Condylar erosion is predictive of painful closed lock of the temporomandibular joint: a magnetic resonance imaging study

**DOI:** 10.1186/s13005-021-00291-1

**Published:** 2021-09-10

**Authors:** Rüdiger Emshoff, Annika Bertram, Linus Hupp, Ansgar Rudisch

**Affiliations:** 1 Orofacial Pain and TMD Unit, University Clinic of Oral and Maxillofacial Surgery, Medical University of Innsbruck, Innsbruck, Austria; 2 Otto von Guericke University of Magdeburg, Magdeburg, Germany; 3 University Clinic of Oral and Maxillofacial Surgery, Medical University of Innsbruck, Innsbruck, Austria; 4 University Clinic of Radiology, Medical University of Innsbruck, Innsbruck, Austria

**Keywords:** Temporomandibular joint, Temporomandibular disorders, Magnetic resonance imaging, Disk displacement, Condylar erosion

## Abstract

**Background:**

To assess whether magnetic resonance imaging (MRI) findings of condylar erosion (CE) are predictive of a specific clinical diagnosis of painful closed lock of the temporomandibular joint (TMJ), and to determine the strength of association between CE and types of internal derangement (ID).

**Methods:**

Based upon sample size estimation, this retrospective paired-design study involved 62 patients, aged between 18 and 67 years. Inclusion criteria were the presence of a unilateral clinical diagnosis of arthralgia coexisting with disk displacement without reduction (‘AR and DDwoR/wLO’), assigned according to the Research Diagnostic Criteria for Temporomandibular Disorders (RDC/TMD) Axis I, and the absence of signs and symptoms of TMJ pain and dysfunction on the contralateral TMJ side. Bilateral sagittal and coronal MR images were obtained to establish the prevalence of CE and TMJ ID types of disk displacement with (DDR) and without reduction (DDNR). Logistic regression analysis was used to compute odds ratios for CE and ID types. Confounding variables adjusted for were age, sex, time since pain onset, pain intensity, and type of ID.

**Results:**

In the regression analysis, the MRI items of DDR (*p* = 0.533) and DDNR (*p* = 0.204) dropped out as nonsignificant in the diagnostic clinical ‘AR and DDwoR/wLO’ group. Significant increases in the risk of ‘AR and DDwoR’ occurred with CE (3.1:1 odds ratio; *p* = 0.026). The presence of CE was significantly related to DDNR (adjusted OR = 43.9; *p* <  0.001).

**Conclusions:**

The data suggest CE as a dominant factor in the definition of painful closed lock of the TMJ, support the view that joint locking needs to be considered as a frequent symptom of osteoarthritis, and emphasize a strong association between the MRI items of CE and DDNR.

## Introduction

Temporomandibular disorders (TMDs) affect the temporomandibular joint (TMJ), masticatory muscles, and associated structures [1]. According to researchers, approximately 65–85% of U. S citizens experience certain TMD symptoms in their lives [2]. An estimated 2% of people with TMD reported to have a limitation in jaw opening, usually referred to as “jaw locking” [3]. Patients may experience prolonged pain and disability, which causes chronic symptoms to become more refractory to traditional medical treatment approaches [4].

Patients with a TMJ closed lock may require additional imaging to determine the nature, location, and extension of the osseous changes, and to detect any associated disk displacements [5, 6]. Currently, magnetic resonance imaging (MRI) is the technique of choice for assessing the pathological conditions of the TMJ [7, 8].

TMJ osteoarthritis (OA) is an inflammatory joint disease that is characterized by deterioration of the articular surfaces and simultaneous remodeling of the underlying bone [9, 10]. Condylar erosion (CE) of the TMJ represents changes in the articular cartilage and the adjacent cortical and subcortical bone [10, 11], which are regarded as a sign of progressive OA [11, 12] and have been associated with characteristic clinical findings, such as pain, joint sounds, and irregular or deviating jaw function [12, 13]. Furthermore, CE is associated with MRI findings of disk displacement [14, 15] and should be adequately addressed in terms of diagnostic and therapeutic management to prevent changes in dentofacial morphology or limited mandibular growth [16].

To the best of the authors’ knowledge, there are no MRI studies available addressing the imaging parameters of TMJ CE and types of internal derangement (ID), i.e., disk displacement with (DDR) and without reduction (DDNR), with a multivariate design in patients with painful TMJ closed lock. Thus, the aim of the present paired design study was to assess whether the MRI findings of CE are predictive of specific clinical diagnosis of painful closed lock of the TMJ, and to determine the strength of the association between the MRI items of CE and types of ID, thereby controlling for confounding variables such as age, sex, time since pain onset, pain intensity, and type of ID.

## Material and methods

### Study design

From a series of 62 consecutive patients, the association between the clinical diagnosis of TMJ arthralgia coexisting with disk displacement without reduction (‘AR and DDwoR/wLO’) and the MRI findings of CE and DDNR, and the relationship between CE and DDNR were analyzed in the MR images of 124 TMJs. There were 56 females and 6 males, aged between 18 and 67 years with a mean age of 35.7 years. Patients with TMJ pain who were referred for treatment by their general practitioner or dentist were eligible for the study. Informed consent was obtained from all participants included in the study. The study followed the Declaration of Helsinki on medical protocols and ethics. Given the retrospective nature of this study, ethical approval of the study was waived by the Ethics Committee of the Medical University of Innsbruck. Criteria for including a TMJ pain patient were (1) the presence of a unilateral TMD diagnosis of ‘AR and DDwoR/wLO’ (AR; self-report of TMJ pain, pain on TMJ palpation, and absence of crepitus; DDwoR/wLO, unassisted opening ≤35 mm and passive stretch ≤4 mm) assigned according to the Research Diagnostic Criteria for Temporomandibular Disorders (RDC/TMD) [17], (2) the absence of signs and symptoms of TMJ pain and dysfunction on the contralateral TMJ side, (3) a pretreatment visual analog scale pain intensity score of > 10 mm, (44) age between 18 and 70, (5) ambulatory status and ability to be treated as an outpatient, and (6) availability for the study schedule.

Criteria for excluding a TMD pain patient were (1) pain attributable to confirmed migraine or to head or neck pain conditions, (2) acute infection or other significant disease of the teeth, ears, eyes, nose, or throat, (3) debilitating physical or mental illness, (4) presence of a collagen vascular disease, (5) history of trauma, and (6) inability to speak or write German.

The evaluation consisted of the collection of basic demographic information, subject self-report measures, history-related questions, and physical examination measures [17]. Each subject completed a visual pain rating assessment of the severity of pain by using a 100-mm visual analog scale, ranging from 0 (no pain) to 100 (very severe pain), on which patients registered the mean pain perceived in the last seven days. This scale has been used extensively in randomized trials and has shown good construct validity in comparison with other pain measures [18, 19].

The study was single-blind, with the clinical records and MR images interpreted by the clinician (RE) and radiologist (AR) independently without knowledge of the results of the other investigation.

### MRI data acquisition

MRI was carried out with a 1.5 T MR scanner (Vision, Siemens AG, Erlangen, Germany) and a dedicated circular-polarized transmit-and-receive TMJ coil. The data were collected on a 252 × 256 matrix with a field of view of 145 mm giving a pixel size of 0.60 × 0.57 mm. With the patient in a supine position, 15 paracoronal and 8 parasagittal slices of each TMJ were obtained using a TSE (turbo spin echo)-PD (proton density) sequence (repetition time of 2800 ms, echo time of 15 ms) and a TIRM (turbo inversion recovery magnitude) sequence (repetition time of 4000 ms, echo time of 30 ms, inversion time of 150 ms) with thin slices of 3 mm. MR images were corrected to the horizontal angulation of the long axis of the condyle.

**Fig. 1:**
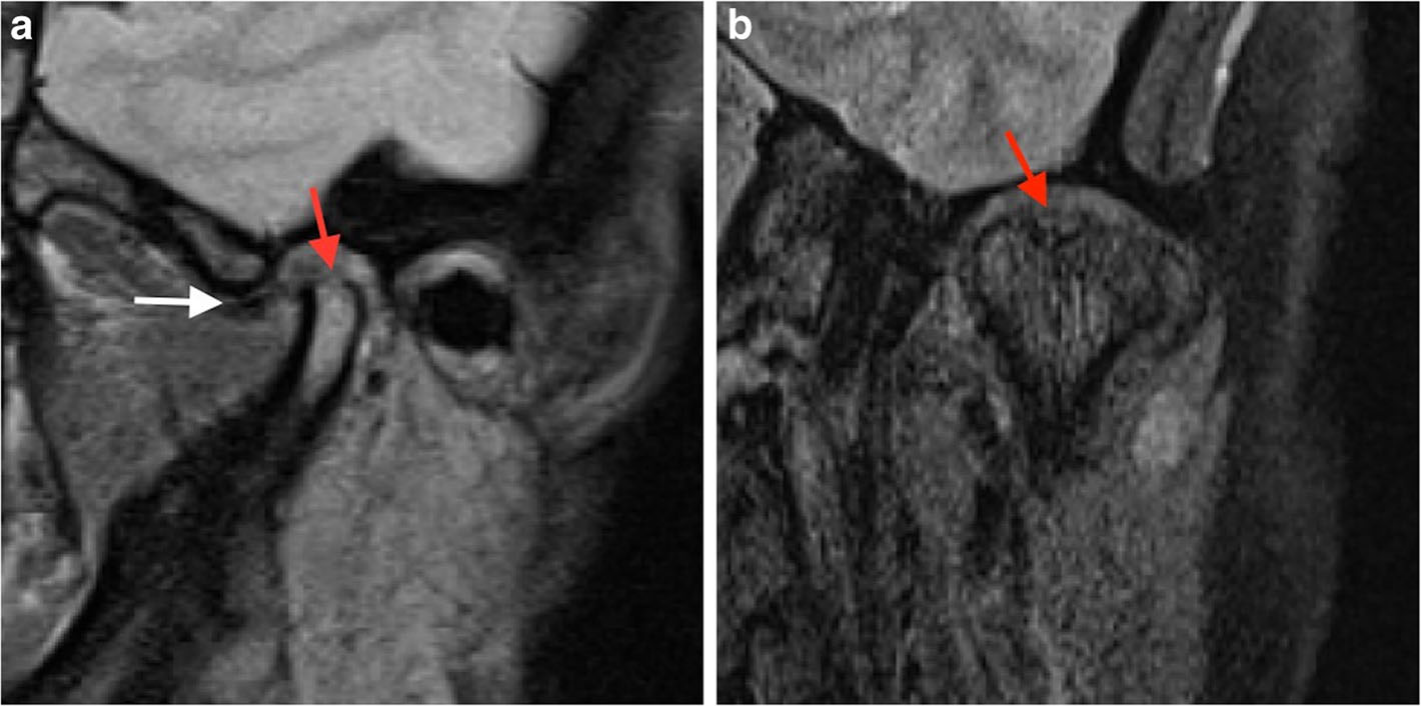
Closed–mouth-related MR images in a 38-year-old female with a 20-month history of left TMJ pain, a TMJ pain-side-related clinical diagnosis of TMJ ‘AR and DDwoR’, and MRI findings of DDNR and CE. Left TMJ with the presence of disk displacement and CE. The sagittal MR image shows the disk displaced anteriorly (white arrow) and the condyle with flattening and erosion (red arrow) (a). The coronal MR image shows the condyle with CE (red arrow) (b)

**Fig. 2:**
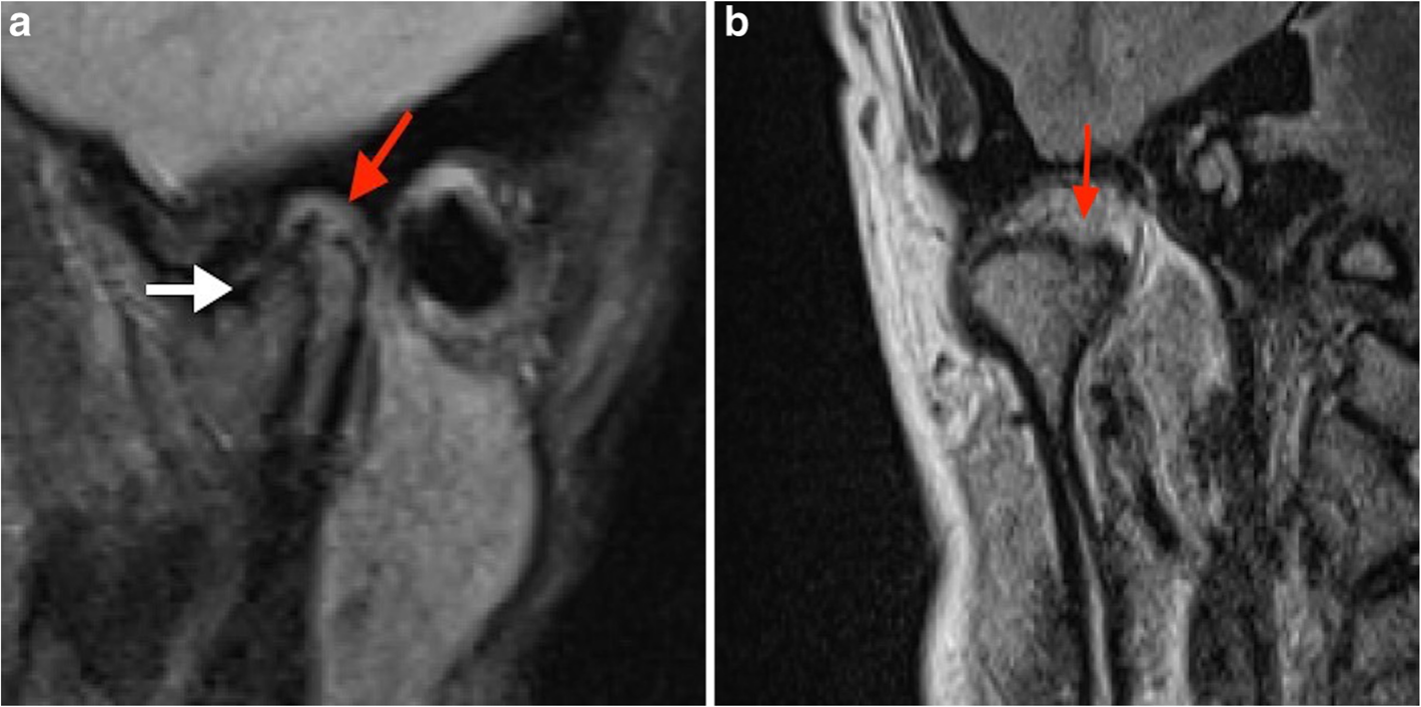
Closed–mouth-related MR images in a 32-year-old female with a 10-year history of right TMJ pain, a TMJ pain-side-related clinical diagnosis of TMJ ‘AR and DDwoR’, and MRI findings of DDNR and CE. Right TMJ with the presence of disk displacement and CE. The sagittal MR image shows the disk displaced anteriorly (white arrow) and the condyle with flattening and erosion (red arrow) (a). The coronal MR image shows the condyle with CE (red arrow) (b)

Each subject received an individual nonferromagnetic intermaxillary device to obtain the different mouth opening positions. Sequential bilateral T1- and T2-weighted images were made at the closed mouth and the respective maximum mouth opening positions. Those T1-weighted images were selected for analysis of the disk-condyle relationship that depicted the disk, condyle, articular eminence, and glenoid fossa. The normal disk position was defined by the location of the posterior band of the disk at the superior or 12 o’clock position relative to the condyle, whereas disk displacement was defined as the posterior band of the disk being in an anterior, anteromedial, anterolateral, medial, or lateral position relative to the superior part of the condyle. The diagnosis of TMJ disk-condyle relationship was categorized as normal or as disk displacement with and without reduction and was defined according to the finding of a closed mouth-related diagnosis of the absence or presence of disk displacement associated with or without an open mouth-related interposition of the disk between the condyle and the articular eminence [20]. MRI findings of osteoarthrosis was defined by the presence of subchondral sclerosis, erosion and osteophytes [15, 21]. MRI diagnosis of condylar erosion was defined as an area of decreased density of cortical bone with or without extension to or below the upper layers of the adjacent subcortical bone (Figs. 1 and 2) [22, 23].

Duplicate determinations were performed on 20 MR images, from which the measurement agreement was calculated by kappa statistics. The intraexaminer reliability for the detection of TMJ DDNR (kappa = 1.00) and TMJ condylar erosion (kappa = 0.90) was high.

### Data analysis

The sample size was established at 62 TMJs (62 cases and 62 controls) by applying G*Power software (version 3.1). The effect size was estimated using sample-based effect size indices based on prevalence estimates derived from previous studies: (1) the prevalence of DDNR was 78.6% among TMJs with DDwoR/wLO and 28.6% among TMJs without pain and dysfunction [24], and (2) the prevalence of CE was 37.2% among TMJs with DDNR and 6.3% among TMJs without an MRI finding of disk displacement [25]. The statistical power was set at 80%, with an alpha error of .05, and an R-squared value of 0.5.

Possible differences in predictor variables between the outcome variables were controlled using independent-samples t test and chi-square analysis. A logistic regression analysis was used to assess the association between the clinical diagnosis of ‘AR and DDwoR/wLO’ and the MRI items of DDNR and CE. Based on previous studies, the variables adjusted for were age (years), sex, time since pain onset, pain intensity, and types of ID [7]. Significance was set at *P* < .05. For the statistical analysis, the PASW 22.0 (SPSS Statistics, IBM, Chicago) package was used.

## Results

The characteristics of TMJs with and without a clinical diagnosis of ‘AR and DDwoR/wLO’ are presented in Table 1. Analysis of side-related data showed that the MRI findings of DDNR (71.0% vs 32.3%) (*p* <  0.001), CE (59.7% vs 21.0%) (*p* <  0.001), and ‘DDNR and CE’ (56.5% vs 17.7%) (*p* < 0.001) were more prevalent in TMJs with a clinical diagnosis of ‘AR and DDwoR/wLO’ than in those without signs and symptoms of pain and dysfunction.

**Table 1:** Sample characteristics of TMJs with and without a clinical diagnosis of ‘arthralgia coexistant with DDwoR’

MRI Variable	TMJ side with ‘arthralgia and DDwoR’ (*n* = 62)	TMJ side without ‘TMJ pain and dysfunction’ (n = 62)	Total (*n* = 124)	*P* Value
Disk displacement				
Disk displacement with reduction (n) (%)	9 (14.5)	23 (37.1)	32 (25.8)	< 0.001^a^
Disk displacement without reduction (n) (%)	44 (71.0)	20 (32.3)	64 (51.6)	< 0.001^a^
Osteoarthrosis				
Subchondral sclerosis (n) (%)	32 (51.6)	31 (50.0)	63 (50.8)	0.857^a^
Osteophyte (n) (%)	19 (30.6)	15 (24.2)	34 (27.5)	0.421^a^
Condylar Erosion (n) (%)	37 (59.7)	13 (21.0)	50 (40.3)	< 0.001^a^
Disk displacement and condylar erosion				
Disk displacement with reduction and condylar erosion (n) (%)	2 (3.2)	2 (3.2)	4 (3.2)	1.000^a^
Disk displacement without reduction and condylar erosion (n) (%)	35 (56.5)	11 (17.7)	46 (37.1)	< 0.001^a^

TMJ: temporomandibular joint, DDwoR: disk displacement without reduction, MRI: magnetic resonance imaging, (%): percent, n: number of TMJs, ^*a*^ based on chi-squared test, P: probability of typ I error

Of the MRI variables considered simultaneously in the logistic regression analysis, the MRI items of DDR (*p* = 0.533), and DDNR (*p* = 0.204) dropped out as nonsignificant in the clinical diagnostic TMJ ‘AR and DDwoR/wLO’ group in comparison with the contralateral TMJ ‘without pain and dysfunction’ group. Significant increases in risk of ‘AR and DDwoR’ occurred with CE (3.1:1 odds ratio; *p* = 0.026) (Table 2).

**Table 2:** Results of regression analysis for TMJ ‘arthralgia coexsistant with DDwoR’

	Analysis	
MRI Variable	OR (95% CI)	P Value
Disk displacement with reduction	0.70 (0.23–2.16)	0.533
Disk displacement without reduction	2.13 (0.66–6.83)	0.204
Condylar erosion	3.14 (1.14–8.62)	0.026

TMJ: temporomandibular joint, DDwoR: disk displacement without reduction, MRI: magnetic resonance imaging, OR: odds ratio, CI*:* confidence interval, P: probability of Typ I error

Analysis of TMJs with and without CE revealed DDNR to be more prevalent in TMJs with CE than in those without CE (92.0% vs 24.3%) (*p* =  0.001), and DDR to be more prevalent in TMJs without CE than in those with CE (37.8% vs 8.0%) (*p* =  0.001). TMJs with CE were associated with a significantly higher level of VAS pain intensity than those without CE (36.4 mm vs 17.3 mm) (*p* <  0.001) (Table 3).

**Table 3:** Sample characteristics of TMJs with and without MRI findings of condylar erosion

Variable	TMJ sides with condylar erosion (*n* = 50)	TMJ sides without condylar erosion (*n* = 74)	Total (n = 124)	P Value
Clinical Variable				
Age (years) (mean ± SD)	40.1 (14.0)	32.8 (12.7)	35.7 (13.6)	0.003^a^
Sex (n) (% female)	46 (92.0)	67 (90.5)	113 (91.1)	0.779^b^
Time since pain onset (weeks) (mean ± SD)	10.3 (11.1)	3.8 (7.2)	6.7 (9.9)	< 0.001^a^
Pain intensity (mm) (mean ± SD)	36.4 (32.2)	17.3 (27.2)	25.1 (30.6)	< 0.001^a^
MRI Variable				
Disk displacement with reduction (n) (%)	4 (8.0)	28 (37.8)	32 (25.8)	0.001^b^
Disk displacement without reduction (n) (%)	46 (92.0)	18 (24.3)	64 (51.6)	0.001^b^

TMJ: temporomandibular joint, MRI: magnetic resonance imaging, SD: standard deviation, (%): percent, n: number of TMJs, ^a^based on independent samples t-test, ^b^based on chi-squared test, probability of typ I error

The odds ratio adjusted for age, sex, time since pain onset, pain intensity, and type of ID that a TMJ with the MRI finding of DDNR might belong to the TMJ CE group was strong (43.9:1) and significant (*p* <  0.001) (Table 4).

**Table 4:** Results of regression analysis for TMJ condylar erosion

	Crude analysis		Adjusted analysis	
MRI Variable	OR (95% CI)	P Value	OR (95% CI)	P Value
Disk displacement with reduction	0.14 (0.05–0.44)	0.001	0.20 (0.06–0.70)	0.012
Disk displacement without reduction	35.78 (11.31–113.16)	< 0.001	43.93 (10.51–183.51)	< 0.001

TMJ: temporomandibular joint, MRI: magnetic resonance imaging, OR: odds ratio, CI: confidence interval, P: probability of typ I error

## Discussion

The results of the present MRI study indicate that TMJs with a clinical RDC/TMD diagnosis of ‘AR and DDwoR/wLO’ are significantly associated with MRI findings of DDNR at a prevalence rate of 71%. This observation compares favorably with those of other authors reporting prevalences of TMJ DDNR in TMJ closed lock instances accounting for 73 to 84% [24, 26–29], while the reported prevalence rates of contralateral asymptomatic TMJs in patients with unilateral TMJ pain have ranged from 19 to 40% [26, 30–34]. These findings may support the concept that nonreducing disks are significantly involved in the clinical presentation of TMJ closed lock. However, considerable attention should be given to the point that other underlying factors may be etiologic in the production of these commonly observed ‘nonreducing disk’ findings, i.e., DDNR may be a highly questionable diagnostic criterion in managing patients with TMJ closed lock, especially for considerations involving surgical procedures.

Concerning the observed MRI prevalence rates of CE (60%) and “DDNR and CE” (57%) in the clinical ‘arthralgia and DDwoR/wLO’ subgroup, the findings may correspond to those of previous research reports describing the prevalences of cone beam computed tomography findings of CE in specific RDC/TMD subgroups of TMJ DDwoR as 25% [35], in TMJ arthralgia as 60% [13], and in TMJ arthritis as 94% [36]; the frequencies in asymptomatic TMJs have been reported to be 6% [21, 35], 22% [37], and 26% [13]. However, in these studies confounding variables were not considered to calculate the respective associations with clinical parameters, i.e., studies failed to take into account variations in the time since pain onset, pain intensity, and MRI findings regarding the ID type.

To the best of our knowledge, this is the first study to provide relative odds for the estimation of painful closed lock of the TMJ in a multivariate design using logistic regression techniques for analysis. This investigation provides a perspective on the contribution of MRI items of CE and ID type to the occurrence of a clinical RDC/TMD diagnosis of ‘AR and DDwoR/wLO’. While the ID types of DDR (0.7:1) and DDNR (2.1:1) did not contribute to the change in risk, a clear definition of the ‘AR and DDwoR/wLO’ group was evident for the MRI variable of CE (3.1:1). Therefore, based on this study, CE may be considered a dominant factor in the definition of painful closed lock of the TMJ. Considering the aspect of arthritic TMJ locking as an underlying mechanism in the etiology of TMJ closed lock, further investigations are indicated to clarify which additional specific OA variables may be associated with an elevated risk for signs and symptoms defining specific groups of TMJ closed lock.

The prevalence of the MRI finding of DDNR in TMJs with CE was 92%, and it carried an increased OR for the TMJ CE group (OR = 43.9:1). Although no data were reported on CE prevalence rates, these observations may compare favorably with the results of another MRI study describing the MRI items of TMJ DDNR and CE as closely related entities (OR = 3.5:1); that is, the TMJs with DDNR appeared to be 3.5 times more likely to have CE than the TMJs without DDNR [21]. However, these results may not be directly comparable, since the latter study failed to use specific TMD subgroups and to adjust for confounding variables such as age, sex, time since pain onset, pain intensity, and type of ID. Further, it must be emphasized that the pathophysiology of CE is unclear and the question whether CE is a sign of progressive OA or an entity related specifically to the onset of DDNR remains unresolved.

The present study asserts that the MRI features of CE may be an essential factor in defining TMJ closed lock patients. Assessing the risk of developing CE should include general and local biochemical factors [9, 11, 38]. Several general factors that have the potential to influence the risk of CE development. They include age, sex, systemic arthritis, and hormonal factors [39, 40]. Mechanical factors comprise of occlusion, disk displacement, trauma, increased friction at the joint, and functional overloading [41–43]. Assessing the additional variables may be considered crucial in defining patients with CE. Unlike a case-control study, a well-controlled cohort study is capable of establishing how specific factors contribute to CE.

Regarding the aspect of prevention and therapy, it is essential not to overemphasize the role of MRI findings of CE and DDNR in order to avoid overlooking other etiological factors that are potentially involved in the production of signs and symptoms characteristic of the clinical ‘AR and DDwoR/wLO’ subgroup. It may be hypothesized that the tendency for TMJs to develop CE in the ‘AR and DDwoR/wLO’ subject group is a consequence of and secondary to mechanical disturbances that may produce an imbalance between anabolic and catabolic processes, progressive degradation of cartilage, and secondary inflammatory components [9, 11]. Moreover, overloading is the main cause of disorders in any synovial joint, including the TMJ [44, 45] and this potentially contributes to the generation of various phenomena, such as adhesive forces, increased friction and shear stress [46, 47]. When lubrication is compromised, various levels of friction are generated between the articular surfaces. Mild friction over a long period of time may contribute to the nonreducing disk process [48], and the articular surface may degenerate due to severe friction, which may also trigger the onset of OA and arthritic TMJ locking [49–51]. From a clinical point of view, therefore, the temporal aspects of pain and dysfunction assessment (i.e., onset, duration, and changes since onset) may become indicative of potential high-risk issues such as the risk of nonreducing disks and/or progressive OA alterations.

The results of this study may suggest the preventive use of MRI in symptomatic cases to differentiate subtypes of degenerative TMJ diseases [16]. These include progressive conditions such as idiopathic condylar resorption (ICR) or juvenile idiopathic arthritis (JIA) [52, 53]. MRI enables the accurate evaluation of TMJ ID and OA changes [8], thereby allowing the detection of CE indicating acute or active alterations. To successfully control these destructive inflammatory conditions and to differentiate between ICR and JIA and erosive TMJ disease [54, 55], early MRI diagnosis of DDNR and associated erosive condylar destruction may become an important factor in terms of prevention and early treatment to prevent changes in dentofacial morphology or limited mandibular growth, leading to facial deformity [16, 52, 55, 56]. Considering that etiology, prognostic aspects, and treatment implications are the main criteria for the utility of diagnostic classifications [57], ongoing research is necessary to determine how well specific findings of CE may demonstrate differences in pathogenesis, treatment, and prognosis.

It is noteworthy that the gold-standard MRI criterion (the 12 o’ clock reference) was used to define normal disk position in the present study [58, 59], and the odds ratios of ID types for predicting a clinical diagnosis of ‘AR and DDwoR/wLO’ established by this criterion were determined. However, disk displacement according to this criterion (the presence of the posterior band of the disk anterior to the 12 o’ clock position) was found in normal aymptomatic volunteers, posing the question of what should be considered an abnormal disk position [8, 60]. More accurate diagnostic operational criteria may be necessary in terms of ‘disease classification’ to identify TMJ disk displacement that is closely linked to the clinical signs and symptoms of particular TMDs, to prevent over- and undertreatment and to achieve a more cost-effective outcome.

The present study may suffer from the lack of evaluation of all aspects of degenerative bony alterations that may affect the articular surfaces of the TMJ. MRI enables the accurate assessment of TMJ OA changes such as flattening, erosion, osteophytes, subchondral bone sclerosis and pseudocysts [16]. The application of MRI in individuals with and without TMJ pain and dysfunction permits the identification, localization, and quantification of these osseous changes, including those that affect the fossa or articular eminence [23]. Ongoing investigations are necessary to determine how well specific TMJ OA changes may show differences in pathogenesis, treatment, and prognosis.

A limitation of this study concerns the aspect that most clinical experience is commonly limited by observer variations, which tend to have a significant impact on the diagnostic process. The possibility of rater bias from the radiologist who assessed the MRI variables must also be considered. Observer performance can be affected by various factors such as training, image quality, and the specific criteria for interpretation. This study used well-defined criteria in the interpretation of MRI variables, and the MR images used were of high quality. The MR images of the TMJ were reported based on intraobserver reliability that was within the accepted limits for a diagnostic study. In contrast, no measurement of interobserver reliability was performed. As a result, overrating may have caused some of the variations in disk displacement and CE reported in this study. Consequently, overestimation of the relevance of these factors to the described disorder groups may have occurred. With regard to the diagnosis of CE, the radiologist may have been influenced by the state of the disk and the other joint components. The most effective way to control for this type of error is to blind the rater in some way. In addition, observer training in the use of specific instruments and the development of specific grading criteria may be considered crucial in protecting observers against rater bias.

Clear directives for diagnosis and treatment of TMJ IDs are often elusive, despite the fact that much research has been done to validate current classification systems, such as the Wilkes Staging System [61], the RDC/TMD [17], and the Diagnostic Criteria (DC) for TMD [62]. Deciding on the terminology that one may use to designate TMJ disorders, it’s up to the clinician to decide wether the diagnostic terminology should be based on clinical manifestations or on structural alterations. The RDC/TMD diagnostic subgroups of ‘arthralgia’ and ‘disk displacement’ represent non-specific clinical manifestations of underlying disease processes, which in fact may be somewhat misleading, because evidence for aetiologic factors is lacking. Basic pathologies encompass inflammation and degeneration in arthritic disorders, and may clinically manifest as pain and biomechanical dysfunction, ie, clicking, intermittent locking, and locking [11, 63]. As diagnosis-making algorithms should provide a basis for effective treatment modalities, and current treatment approaches mainly focus on influencing the pathologic changes without addressing positional changes of the disk [63], the RDC/DC terminology used in the ‘disk displacement’ domain may confuse diagnosis and ultimately treatment and management of these patients.

## Conclusions

The present data suggest that CE is a dominant factor in the definition of painful closed lock of the TMJ, support the view that joint locking needs to be considered as a frequent symptom of OA, and emphasize a strong association between the MRI items of CE and DDNR.

## Data Availability

Due to the nature of this research, participants of this study did not agree for their data to be shared publicly, so supporting data is not available.

## Abbreviations

CE: condylar erosion
DDNR: disk displacement without reduction
DDR: disk displacement with reduction
DDwoR: disk displacement without reduction
DDwR: disk displacement with reduction
ID: internal derangement
MRI: magnetic resonance imaging
OA: osteoarthritis
OR: odds ratio
RDC/TMD: Research Diagnostic Criteria for Temporomandibular Disorders
TMJ: temporomandibular joint
